# Reflections on Transitioning a Regional Teaching Programme from Online to In-Person Delivery

**DOI:** 10.1192/bjo.2026.11326

**Published:** 2026-06-30

**Authors:** Estela da Rosa Reckziegel, Joshua Withers, Jonathan Monk-Cunliffe, Daniel McNamara

**Affiliations:** Avon & Wiltshire Mental Health Partnership, Bristol, United Kingdom

## Abstract

**Aims:**

Online teaching is common practice in postgraduate medical education. For the past three years our trust has provided an optional postgraduate education programme in psychiatry to GPs and foundation doctors. This has been run online due to perceptions of increased learner convenience, reduced time-cost for busy clinician teachers and reduced financial cost to the organisation. However, the programme’s teaching faculty observed little learner engagement in interactive learning during online sessions and were concerned about the impact of this on the teaching and learning experience. It was postulated that in-person sessions might increase group proximity, cohesiveness and psychological safety leading to a more interactive learning and teaching experience and increased learner and teacher satisfaction.

**Methods:**

We reorganized our regional teaching programme and changed 6 online sessions to one half educational day with 3 sessions, and a full day with the remaining 4 sessions. We delivered the first of these sessions in early January 2026. Session content included psychiatric emergencies, communication skills, and comparisons between psychiatry and other specialties.

**Results:**

7 out of 52 learners attended the first session, the majority of whom were based in Bristol placements. This attendance was lower than that observed for previous online sessions (between 8 and 15 attendees out of 52 invited) and could reflect commuting and obtaining time away from clinical duties. In contrast, online sessions had previously been attended bytrainees from across the region, raising potential equity and accessibility considerations. Despite the lower attendance, formal feedback from attendees was uniformly positive. Informal feedback indicated a preference for in-person teaching, with learners reporting improved concentration and greater ease of interaction with both faculty and peers. Teachers had the impression learners engaged more in the sessions in contrast to previous online ones, and had higher satisfaction with the format, which may have implications for teacher motivation and retention.

**Conclusion:**

We have recently changed our GP and foundation doctors teaching programme from online to in-person sessions due to concerns about low engagement within sessions. Our early reflections include a better perceived experience from learners and teachers, although difficulties in attending sessions could limit success in the future. We have demonstrated satisfaction with returning to in-person teaching, and will continue to assess learners’ responses and feedback.

